# Case Report: Relapsed alveolar rhabdomyosarcoma treated with abemaciclib, temozolomide, and irinotecan in the JPCS study

**DOI:** 10.3389/fonc.2025.1637177

**Published:** 2025-08-27

**Authors:** Antonio Juan Ribelles, Nuria Benavent, Daniel Sanchez Mateos, Celine Pitou, Yanhong Zhou, Molly C. Hardebeck, Holly Knoderer, Adela Cañete

**Affiliations:** ^1^ Pediatric Oncology and Hematology Unit, University Hospital and Polytechnic La Fe, Valencia, Spain; ^2^ Pediatric Oncology and Hematology Unit, La Fe Health Research Institute, Valencia, Spain; ^3^ Pediatric Radiology Unit, University Hospital and Polytechnic La Fe, Valencia, Spain; ^4^ Eli Lilly and Company, Indianapolis, IN, United States

**Keywords:** abemaciclib, CDK4/6 inhibitor, rhabdomyosarcoma, JPCS, pediatric, case report

## Abstract

**Background:**

Cyclin-dependent kinase (CDK) 4 and CDK6 play fundamental roles in cell cycle progression. The CDK4/6 inhibitor abemaciclib, in combination with temozolomide and irinotecan, was evaluated in pediatric and young adult patients with relapsed/refractory solid tumors in the phase 1b dose-escalation study, JPCS Part A (NCT04238819). This case report describes the notable results of a patient with relapsed alveolar rhabdomyosarcoma (ARMS) who experienced a prolonged complete response.

**Case presentation:**

An 8-year-old White male was initially diagnosed with stage IV ARMS with *PAX3*-*FOXO1* fusion. Molecular characterization following a fourth relapse revealed *CDK4*, *ERBB3*, *GLI1*, *MYCN*, and *FGFR4* amplifications and *MYCN* mutation. After five relapses, the patient enrolled in JPCS Part A and received abemaciclib (55 mg/m^2^ twice daily continuously) in combination with temozolomide (100 mg/m^2^ daily) and irinotecan (50 mg/m^2^ daily) on days 1 to 5 of 21-day cycles. The patient received 12 cycles of the triplet combination, followed by 23 additional cycles of abemaciclib monotherapy. Complete response (CR) was achieved in less than 3 months, with a duration of response (DOR) of 22.6 months and progression-free survival (PFS) of 23.7 months. The study treatment was well tolerated.

**Conclusion:**

CDK4/6 inhibition with abemaciclib in combination with temozolomide and irinotecan provided a durable response in a patient with heavily pretreated ARMS. Additional studies may be warranted to further understand the role of CDK4/6 inhibitors for treatment of ARMS.

## 1 Introduction

Rhabdomyosarcoma (RMS) is a malignant tumor of skeletal myoblast-like cells and is the most common pediatric soft-tissue sarcoma, with an incidence of approximately 4.5 cases per million children/adolescents per year (1, 2). Although 5-year overall survival exceeds 70%, approximately one-third of patients experience progression/relapse within 13 months of diagnosis, and 5-year post-relapse survival remains approximately 24% (3, 4). Treatment for relapsed disease typically consists of multi-agent chemotherapy combinations with or without surgery and radiotherapy and often results in minimal benefit, indicating an urgent need for novel therapeutics, especially for patients with multiple relapses (3).

Two major RMS subtypes exist—*PAX3/7*-*FOXO1* fusion-negative, also known as embryonal RMS, and *PAX3/7*-*FOXO1* fusion-positive, also known as alveolar RMS (ARMS), each with a unique array of molecular alterations (5). The *PAX3/7*-*FOXO1* fusion-negative subtype commonly exhibits loss of heterozygosity at the 11p15 locus, which contains the *IGF-II* gene and often has *FGFR1* and *NRAS* mutations (6). In contrast, the *PAX3/7*-*FOXO1* fusion-positive subtype, the most aggressive RMS subtype, is characterized by a translocation between the *PAX3/7* and *FOXO1* genes and amplification of *MYCN* and cyclin-dependent kinase (CDK) 4 (7, 8). Moreover, CDK4 is amplified in approximately 25% of *PAX3*-*FOXO1* cases and approximately 4% of *PAX7*-*FOXO1* cases (9). Other aberrations in the CDK4/6 pathway have been reported, including the homozygous deletion of *CDKN2A/B* and amplification of *CCND2* and *CCND3* (10–12). CDK4/6 pathway alterations may render fusion-positive cells vulnerable to targeted treatment with a CDK4/6 inhibitor.

Abemaciclib is an oral, potent, and selective CDK4/6 inhibitor approved for certain types of early stage and metastatic breast cancers (13–15). Abemaciclib is a unique CDK4/6 inhibitor due to its continuous dosing schedule, greater relative potency to CDK4 than CDK6, and its ability to penetrate the central nervous system (16–19). Abemaciclib blocks CDK4/6 from phosphorylating the retinoblastoma tumor suppressor, which results in G1 arrest and inhibition of cancer cell growth (16). The phase 1b study, I3Y-MC-JPCS (JPCS), evaluated abemaciclib in combination with temozolomide and irinotecan in pediatric and young adult patients with relapsed/refractory solid tumors. This case report examines the notable results of an adolescent patient enrolled in the JPCS study with relapsed ARMS who achieved a durable complete response.

## 2 Case presentation

**Figure 1:**
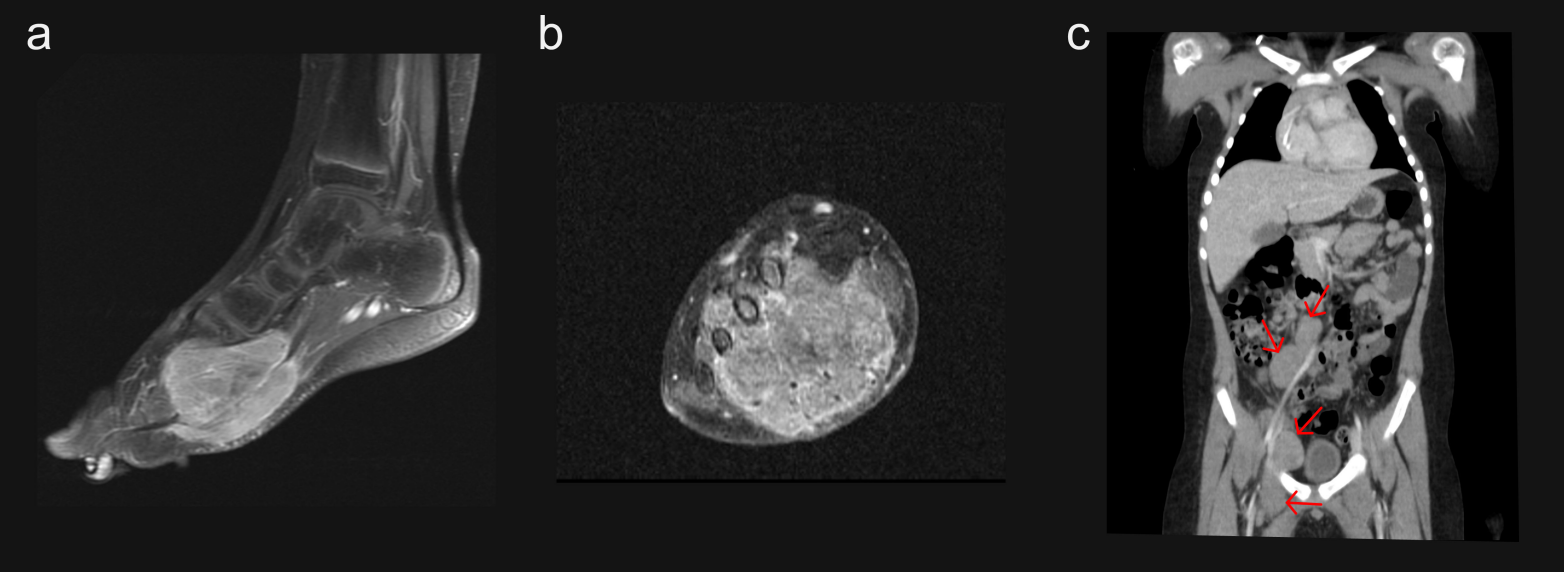
Primary tumors at initial diagnosis. **(a)** MRI sagittal plane of the right foot; **(b)** MRI coronal plane of the right foot; **(c)** Abdominal CT coronal plane showing inguinal and retroperitoneal malignant lymph nodes. CT, computed tomography; MRI, magnetic resonance imaging.

An 8-year-old White male was diagnosed with stage IV *PAX3*-*FOXO1* fusion-positive ARMS of the right foot and multiple lymph nodes with confirmed disease (**Figure 1**). Tumor biomarker analysis following the fourth relapse revealed a *MYCN* mutation (c.131C>T resulting in p.Pro44Leu amino acid alteration) and amplification of *CDK4*, *MYCN*, *ERBB3*, *GLI1*, and *FGFR4*. No *RB1* alterations were detected. The patient had received five treatment regimens over approximately 7 years (**Table 1**). During this time, the patient underwent multiple surgeries and four courses of radiotherapy targeting lesions in the lower extremity, mesenteric nodes, muscle, and abdominal-pelvic regions.

**Table 1:** Prior therapies and outcomes.

Regimen number	Treatment	Approximate time on treatment (months)	Best response	Approximate time off therapy until next relapse (months)
**1**	Ifosfamide, vincristine, dactinomycin, doxorubicin, cyclophosphamide, vinorelbine	13^a^	CR	7^c^
**2**	Vincristine, irinotecan	10	CR	24^d,e^
**3**	Regorafenib, vincristine, irinotecan	2	SD	Progressed on treatment
**4**	Topotecan, carboplatin, cyclophosphamide	14^b^	CR	4^f^
**5**	Trabectedin	5	CR	6^g^

CR, complete response; SD, stable disease.

a Ifosfamide, vincristine, dactinomycin, and doxorubicin were given for approximately 6 months, followed by a 2-month break, and then cyclophosphamide and vinorelbine were given as maintenance therapy for approximately 5 months.

b Topotecan and carboplatin were given for approximately 5 months and 2 months, respectively, followed by a 2-month break, and then cyclophosphamide was given as maintenance therapy for approximately 7 months.

c Post regimen 1, patient experienced primary resection (tibia) followed by retroperitoneal lymph node, iliac lymph node and right femoral-inguinal lymph nodal radiation.

d Prior to regimen 2, patient experienced mesenteric lymph nodal resection and appendix removal.

e During regimen 2, patient also experienced abdominal radiation.

f During regimen 4, patient also underwent soleus muscle resection followed by radiation.

g Prior to regimen 5, patient experienced popliteal lymph nodal resection.

h During regimen 5, patient also experienced popliteal lymph node radiation.

**Figure 2:**
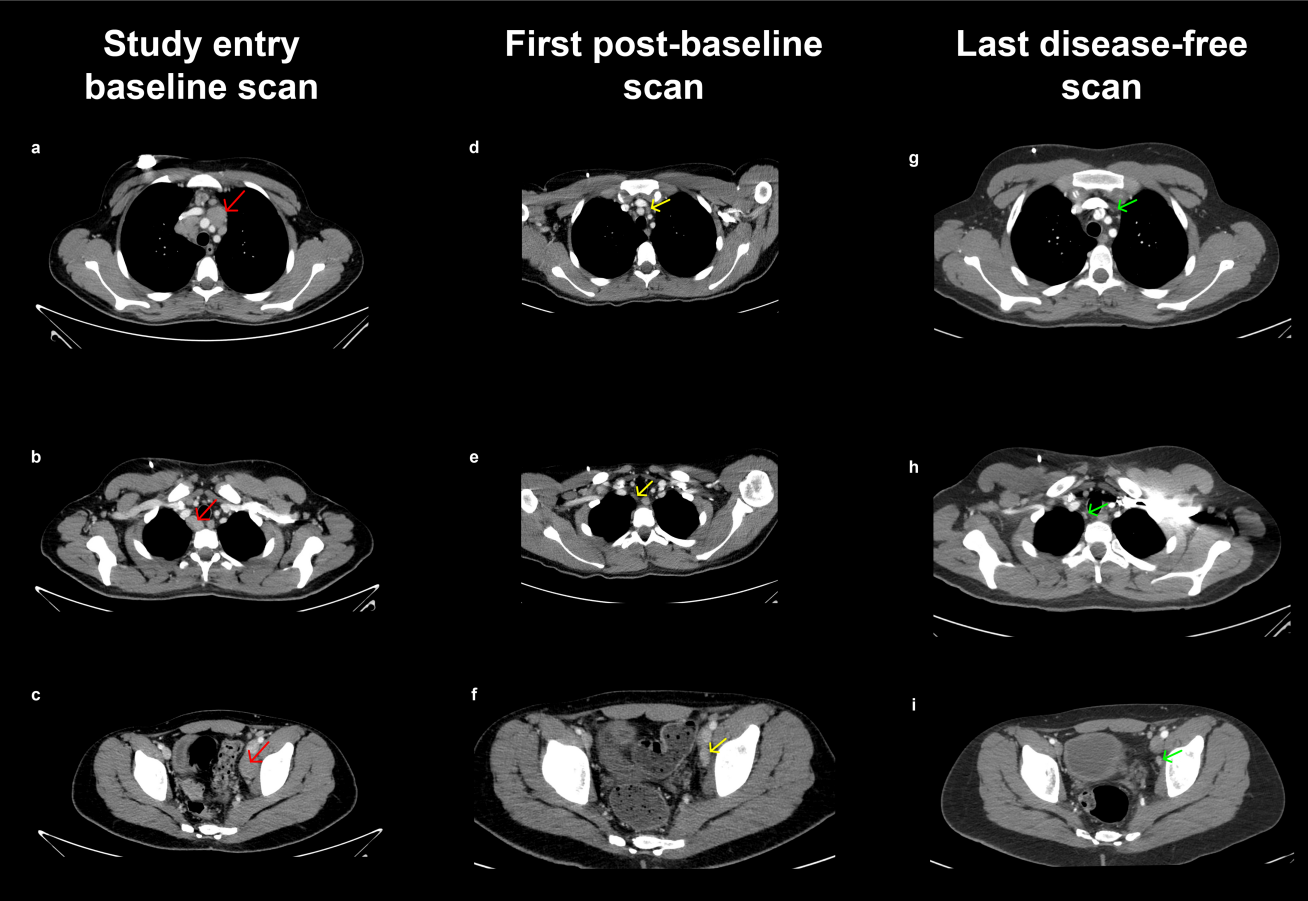
Tumors at JPCS study entry, first post-baseline scan, and last disease-free scan. **(a–c)** CT scan of the chest, abdomen, and pelvis with intravenous contrast at baseline. Multiple tumor lymphadenopathies were identified: bilateral supraclavicular, mediastinal, and an adenopathic conglomerate in the left iliac axis. **(d–f)** First post-baseline CT scan of the chest, abdomen, and pelvis with intravenous contrast. A significant reduction in the size and number of bilateral supraclavicular, mediastinal, retroperitoneal, and left iliac axis adenopathy is observed compared to the previous scan. **(g–i)** Last disease-free CT scan of the chest, abdomen, and pelvis with intravenous contrast showing complete resolution of tumor lymphadenopathy initially identified as target lesions. CT, computed tomography.

After the fifth relapse, the patient enrolled in JPCS, a phase 1b trial with a 3 + 3 dose-escalation design, for pediatric and young adult patients with relapsed/refractory solid tumors. The patient was assigned to abemaciclib (55 mg/m^2^ twice daily [BID continuously]) in combination with both temozolomide (100 mg/m^2^ daily) and irinotecan (50 mg/m^2^ daily) on days 1 to 5 of 21-day cycles. No preexisting conditions were reported. The disease sites at study entry included five target tumors (located in the supraclavicular lymph node, mediastinal lymph node ×2, and iliac lymph node ×2) and four non-target tumors (located in the supraclavicular lymph node, mediastinal lymph node, iliac lymph node, and retroperitoneum; **Figures 2a–c**). The patient received 35 cycles of abemaciclib, the first 12 in combination with temozolomide and irinotecan (discontinuation of chemotherapy was permitted after cycle 12 per protocol). Treatment compliance ranged from 98% to 100% for all three study drugs.

**Figure 3:**
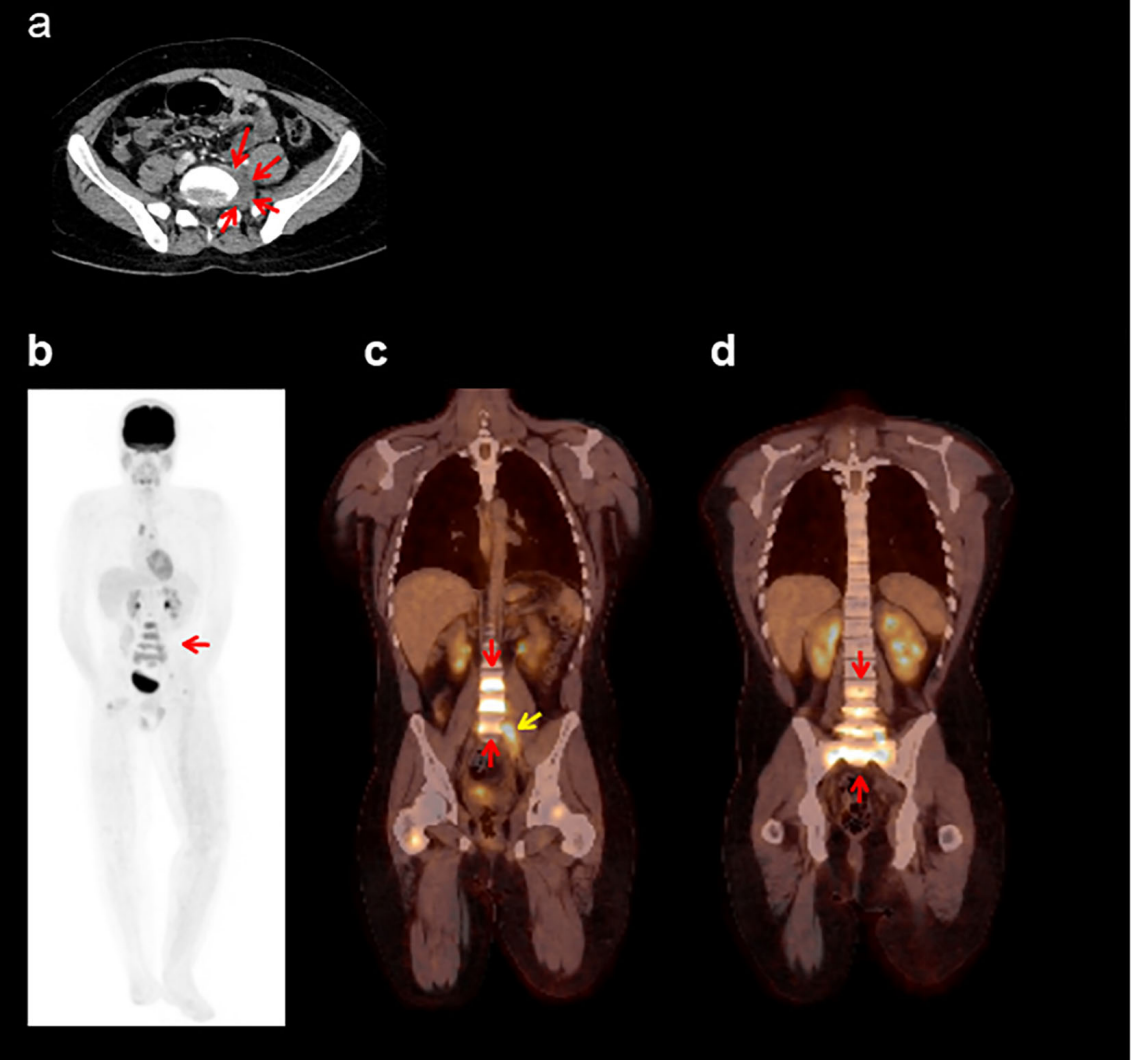
Tumor at progressive disease. **(a)** CT scan indicating progressive disease in the left paravertebral soft tissue mass (red arrows). **(b–d)** Images correspond to the PET-CT study at progressive disease—MIP image **(b)** and coronal fusion images **(c, d)**—which show a diffuse increase in metabolism in the lumbosacral vertebrae, encompassing the entirety of the vertebral bodies (red arrows), as well as hypermetabolism associated with the soft tissue mass in the left paravertebral region at L5 (yellow arrow). CT, computed tomography; MIP, maximum intensity projection; PET, positron emission tomography.

Computed tomography scans were obtained after cycle 2, cycle 4, and every third cycle thereafter. Partial response was achieved at the first scan (**Figures 2d–f**), followed by complete response at day 78. The last disease-free scan is shown in **Figures 2g–i**. The patient had ongoing benefit for 22.6 months until disease progression when five new tumors (left hilar adenopathy, lumbar and sacral spine, L5 vertebra, D8 vertebra, and femurs) were identified (**Figure 3**). Progression-free survival was 23.7 months.

The patient tolerated study treatment well and experienced no dose-limiting toxicities or serious adverse events. The most frequent treatment-related adverse events were thrombocytopenia, diarrhea, dizziness, nausea, vomiting (all with a maximum severity of grade 1), and neutropenia (maximum severity of grade 3). Abemaciclib was withheld for 3 days due to an adverse event of grade 3 aspartate aminotransferase increase that was deemed unrelated to treatment, and there were no dose reductions. There were no dose modifications of temozolomide or irinotecan.

Pharmacokinetics assessments demonstrated that abemaciclib exposure and that of its M2 and M20 metabolites as well as temozolomide, irinotecan, and its metabolite SN-38 were consistent with that in other patients who received the same dose (data on file (20);).

## 3 Discussion

ARMS is a recalcitrant disease with rapid spreading, dismal therapeutic response, and high relapse rates (21). Given the poor prognosis, new targeted therapeutic approaches are needed. This case report highlights the notable results in a patient with relapsed ARMS who was treated with temozolomide and irinotecan in combination with the CDK4/6 inhibitor, abemaciclib.

Deregulation of the CDK4/6 pathway has been reported in ARMS, although its contribution to oncogenesis and disease progression is not fully understood (21). The primary CDK4/6 pathway alteration in ARMS is *CDK4* amplification, which is present in about 25% of *PAX3–FOXO1*-positive tumors and approximately 4% of *PAX7–FOXO1*-positive tumors (9). The mechanism of *CDK4* amplification and overexpression is likely via amplification of the 12q13-q14 amplicon (21, 22). Based on the frequent *CDK4* alterations, abemaciclib may serve as a rational therapeutic approach to counteract the resulting cell cycle abnormalities and inhibit ARMS progression.

The case presented herein demonstrates a prolonged response to treatment with abemaciclib and standard chemotherapy in a patient with fifth relapse, stage IV, *CDK4*-amplified, *PAX3*-*FOXO1* fusion-positive ARMS. The patient discontinued chemotherapy after 12 cycles and continued abemaciclib monotherapy for 23 additional cycles, which suggests that abemaciclib significantly contributed to the sustained complete response.

In addition to CDK4 amplification, this patient’s tumor also harbored FGFR4 amplification, which has been associated with aggressive RMS behavior and therapeutic resistance (23). While the contribution of FGFR4 to the observed response is unclear, its presence highlights the complexity of molecular alterations in ARMS and the potential need to consider co-alterations in future studies (24).

Recent studies in other CDK4-amplified sarcomas, such as dedifferentiated liposarcoma and synovial sarcoma, have demonstrated synergistic antitumor effects and prognostic significance of CDK4/6 inhibition (25, 26). These findings further support the broader relevance of investigating CDK4/6 across soft tissue sarcomas and further support exploration of this therapeutic strategy in ARMS.

The findings in this case contrast with the results of studies of other CDK4/6 inhibitors as monotherapy or in combination, which were less promising in a limited number of patients with ARMS-containing alterations in the CDK4/6 pathway (27, 28). Likewise, two other patients with RMS (unknown subtype) in the JPCS Part A study did not achieve durable responses. Thus, the molecular mechanisms rendering sensitivity in some cases and resistance in other cases remain elusive.

Preclinical studies have postulated mechanisms to explain CDK4/6 inhibitor response. A study evaluating fusion-positive RMS suggested that CDK4/6 inhibitor sensitivity depends on the level of *CDK4* amplification; specifically, all fusion-positive models evaluated exhibited sensitivity, but models with lower *CDK4* amplification (though higher than no amplification) were more sensitive than those with higher CDK4 levels (9). The researchers hypothesized that cyclin D1 expression may saturate CDK4 and limit the effects of *CDK4* overexpression (9). Furthermore, preclinical evidence has linked *Rb*1 loss to ARMS progression and demonstrated that downregulation of pRb expression leads to CDK4/6 inhibitor resistance (29). Thus, effective CDK4/6 inhibition likely hinges not only on appropriate CDK4 levels but also on enduring Rb functionality.

This case report is inherently limited by its single-patient nature which restricts the generalizability of the findings. While the prolonged complete response is notable, broader conclusions regarding the efficacy of CDK4/6 inhibition in ARMS require validation through larger controlled studies.

The evidence presented in this case report strengthens the argument that CDK4/6 may play an important role in the pathogenesis of *CDK4*-amplified ARMS and that abemaciclib in combination with temozolomide and irinotecan may abrogate tumor growth and improve outcomes. However, this evaluation is limited to a single patient, and we do not yet fully understand the biological or molecular basis for this patient’s exceptional initial response or the factors that led to eventual disease progression. It should be noted that this patient had achieved complete responses when treated with prior regimens and thus may have a unique molecular profile yielding favorable outcomes. Nonetheless, a nearly 2-year treatment response for fifth relapse ARMS is remarkable. These results provide the first insights into the clinical activity of abemaciclib, temozolomide, and irinotecan in *CDK4*-amplified ARMS, and additional studies may be warranted to further understand the role of *CDK4/6* alterations in ARMS. Further research could explore biomarker-guided trials to identify benefit of CDK4/6 inhibition and evaluate this combination in prospective studies across molecularly defined RMS subtypes. Such studies could clarify the mechanisms of response and resistance and inform patient selection strategies.

## 4 Conclusion

Novel treatments that exploit targetable alterations are needed to improve outcomes for relapsed ARMS. This case report describes an adolescent male with fifth relapse, *CDK4*-amplified, *PAX3*-*FOXO1* fusion-positive ARMS who had a remarkable response to treatment with the CDK4/6 inhibitor abemaciclib in combination with temozolomide and irinotecan. These results underscore the importance of exploring CDK4/6 inhibition as a potential treatment strategy for ARMS.

## Data Availability

Eli Lilly and Company provides access to all individual participant data collected during the trial, after anonymization, with the exception of pharmacokinetic or genetic data. Data are available to request 6 months after the indication studied has been approved in the United States and European Union and after primary publication acceptance, whichever is later. No expiration date of data requests is currently set once data are made available. Access is provided after a proposal has been approved by an independent review committee identified for this purpose and after receipt of a signed data sharing agreement. Data and documents, including the study protocol, statistical analysis plan, clinical study report, blank or annotated case report forms, will be provided in a secure data sharing environment. For details on submitting a request, see the instructions provided at www.vivli.org.
